# Obesity as risk factor for subtypes of breast cancer: results from a prospective cohort study

**DOI:** 10.1186/s12885-018-4548-6

**Published:** 2018-05-31

**Authors:** Cina J. Nattenmüller, Mark Kriegsmann, Disorn Sookthai, Renée Turzanski Fortner, Annika Steffen, Britta Walter, Theron Johnson, Jutta Kneisel, Verena Katzke, Manuela Bergmann, Hans Peter Sinn, Peter Schirmacher, Esther Herpel, Heiner Boeing, Rudolf Kaaks, Tilman Kühn

**Affiliations:** 1 Division of Cancer Epidemiology, German Cancer Research Center (DKFZ), Im Neuenheimer Feld 280, Heidelberg, Germany; 2 Institute of Pathology, University Hospital Heidelberg, Heidelberg, Germany; 3 Department of Epidemiology, German Institute of Human Nutrition (DIfE) Postdam-Rehbrücke, Nuthetal, Germany; 4 Tissue Bank of the National Center for Tumor Diseases (NCT), Heidelberg, Germany

**Keywords:** Breast cancer, Obesity, Tumor subtypes, Estrogen receptor, Ki-67, p53, Bcl-2

## Abstract

**Background:**

Earlier epidemiological studies indicate that associations between obesity and breast cancer risk may not only depend on menopausal status and use of exogenous hormones, but might also differ by tumor subtype. Here, we evaluated whether obesity is differentially associated with the risk of breast tumor subtypes, as defined by 6 immunohistochemical markers (ER, PR, HER2, Ki67, Bcl-2 and p53, separately and combined), in the prospective EPIC-Germany Study (*n* = 27,012).

**Methods:**

Formalin-fixed and paraffin-embedded (FFPE) tumor tissues of 657 incident breast cancer cases were used for histopathological analyses. Associations between BMI and breast cancer risk across subtypes were evaluated by multivariable Cox regression models stratified by menopausal status and hormone therapy (HT) use.

**Results:**

Among postmenopausal non-users of HT, higher BMI was significantly associated with an increased risk of less aggressive, i.e. ER+, PR+, HER2-, Ki67_low_, Bcl-2+ and p53- tumors (HR per 5 kg/m^2^: 1.44 [1.10, 1.90], *p* = 0.009), but not with risk of more aggressive tumor subtypes. Among postmenopausal users of HT, BMI was significantly inversely associated with less aggressive tumors (HR per 5 kg/m^2^: 0.68 [0.50, 0.94], *p* = 0.018). Finally, among pre- and perimenopausal women, Cox regression models did not reveal significant linear associations between BMI and risk of any tumor subtype, although analyses by BMI tertiles showed a significantly lower risk of less aggressive tumors for women in the highest tertile (HR: 0.55 [0.33, 0.93]).

**Conclusion:**

Overall, our results suggest that obesity is related to risk of breast tumors with lower aggressiveness, a finding that requires replication in larger-scale analyses of pooled prospective data.

**Electronic supplementary material:**

The online version of this article (10.1186/s12885-018-4548-6) contains supplementary material, which is available to authorized users.

## Background

Associations between etiological factors and cancer risk have been shown to be differential across molecular tumor subtypes in earlier epidemiological studies [1, 2]. With respect to relationships between anthropometric factors and breast cancer risk, there is evidence to suggest that obesity, as measured by body mass index (BMI), increases the risk of estrogen receptor positive (ER+) rather than ER- breast tumors in postmenopausal women [3–5]. Moreover, it has been proposed that obesity is related to more slowly proliferating tumors, as defined by low expression of the Ki67 protein in tumor cells [5]. Thus, mechanisms to link obesity with breast cancer, especially altered estrogen and Insulin-like growth factor 1 (IGF-1) signaling [6], could drive overall less aggressive tumors with a distinct molecular profile. However, despite the notion that a better understanding of risk factor associations with tumor subtypes is needed to improve personalized medicine and prevention [1], prospective data on the relationship between anthropometric parameters and the risks of breast cancer by subtypes beyond those defined by hormone receptor status are sparse [2].

The aim of the present study was to examine the associations between obesity with breast cancer risk across more refined tumor subtypes. For this purpose, we assessed six well-established immunohistochemical markers (ER, PR, HER2, Ki67, Bcl-2 and p53) in tumor samples of breast cancer cases from the prospective European Prospective Investigation into Cancer and Nutrition (EPIC)-Germany Study. We hypothesized that obesity would be particularly related to the development of less aggressive tumors (i.e. ER+, PR+, HER2-, Ki67_low_, Bcl-2+ and p53- tumors).

## Methods

### Study population

EPIC is a multi-center prospective cohort study with more than 500,000 participants across Europe. In Germany, 53,088 participants (30,270 women) in the age range between 35 and 65 years were recruited at the study centers in the cities of Heidelberg and Potsdam between 1994 and 1998 [7, 8]. At baseline, anthropometric measurements were carried out by trained personnel, and data on diet, physical activity, smoking, alcohol consumption, medication use, reproductive factors and socio-economic status were obtained [7].

Incident cases of breast cancer were either self-reported during follow-up or derived from cancer registries. Each case was validated by a study physician using the information given by the patient’s treating physicians and hospitals. Overall, 1095 cases of primary breast cancer had occurred until Dec 31st 2010, the closure date for the present analyses. After exclusion of prevalent cases of cancer (*n* = 1669), individuals lost to follow-up (*n* = 947), individuals with unclear breast cancer status (*n* = 23), individuals with missing covariate information (*n* = 181), and incident cases without tumor blocks (*n* = 438) from the EPIC-Germany cohort, the study population for the present analyses comprised 27,012 women (Additional file 1: Figure S1).

### Laboratory methods

Formalin-fixed paraffin-embedded (FFPE) tumor tissue material was available for a total of 657 cases (60.0%). There were no significant statistical differences regarding age, reproductive factors and lifestyle factors between these cases and those for which no tumor blocks were available, even though there were slightly more in situ and grade I tumors in the latter group (Additional file 2: Table S1). A board-certified senior pathologist (E.H.) selected representative tumor areas to construct tissue microarrays (TMA) on a hematoxylin and eosin stained slide of each tumor block. A TMA machine (AlphaMetrix Biotech, Roedermark, Germany) was used to extract tandem 1 mm cylindrical core samples. IHC staining was carried out using antibodies routinely employed for diagnostic purposes (Additional file 2: Table S2) and an immunostaining device (DAKO, Techmate 500plus). All TMA slides were examined by at least one pathologist (E.H., M.K.) with special expertise in breast cancer pathology. In case of a discrepancy between the scores derived from the first and second core of the same patient, the pathologists re-examined both cores and made a final decision. Whenever TMA analysis did not yield a conclusive result for a marker, it was assigned a missing value (ER: 2.0%; PR: 2.7%; HER2: 1.7%; Ki67: 6.1%; Bcl-2: 4.1%; p53: 6.7%). Tumors were categorized as ER positive/negative and PR positive/negative using the Allred Score [9]. HER2 was determined according to staining pattern and intensity, and scored as negative (0 and 1+) or positive (2+ and 3+) [10]. Ki67 proliferation activity was scored by percentage of positive tumor nuclei (< 20%: low proliferative activity; ≥20%: high proliferative activity) [11]. Bcl-2 was scored as negative if less than 10% of the cells were positive and staining intensity was weak, otherwise Bcl-2 was scored as positive [12]. Cases with more than 10% of cells stained were rated p53 positive, the remaining cases were rated p53 negative, as in most previous studies using this antigen [13]. Categorization of subtypes was based on visual estimation counting at least 100 tumor cells.

### Statistical analyses

Relationships between BMI at recruitment and breast cancer risk were evaluated separately among 1) women, who were pre- or perimenopausal at baseline 2) women, who were postmenopausal at baseline and used hormone therapy (HT), and 3) women, who were postmenopausal at baseline and did not use HT, as differential risk associations with BMI across these subgroups have been reported [14, 15]. Statistical analyses on breast cancer risk by tumor subtype were carried out using multivariable Cox proportional hazards regression analyses to estimate hazard ratios (HR) and 95% confidence intervals (CI) across tertiles of BMI (created based on data of the full cohort), with age as the underlying time scale. All models were adjusted for height (continuous), number of full-term pregnancies (continuous), educational level (university degree vs. no university degree), smoking status (never, former, current), and study center (Heidelberg, Potsdam). Analyses among pre- and perimenopausal women were further adjusted for current use of oral contraceptives. The inclusion of other potential confounders (alcohol consumption, breast feeding, age at menarche, age at first pregnancy) only marginally affected risk associations and were not included in final Cox regression models.

Linear trends were estimated by entering BMI as a continuous term into the same model rescaling HRs to reflect a 5 kg/m^2^ increase. Observations were left-truncated and censored at end of follow-up, death, or cancer diagnosis, whichever occurred first. In order to assess patterns of IHC markers, unsupervised hierarchical clustering was used to group cancer cases according to the similarity / dissimilarity of the IHC staining results for ER, PR, HER2, Ki67, Bcl-2, and p53, as previously published [16, 17]. In addition to BMI, we evaluated waist circumference and hip circumference as anthropometric markers of obesity in relation to breast cancer risk. Heterogeneity in associations between anthropometric factors and breast cancer risk across subtypes was tested for using a competing risk framework, as proposed by Wang et al. [18]. As the evidence on associations between BMI and in situ breast tumors is not consistent [19, 20], we decided to exclude cases of in situ tumors in sensitivity analyses. All statistical analyses were carried out using SAS, version 9.4 (SAS Institute, Cary, NC, USA). For unsupervised hierarchical clustering and for the generation of a dendogram / heat map to visualize clusters of tumor markers we used the *d3heatmap* package in R [21].

## Results

### Characteristics of the study population

The analytical cohort for the present analyses comprised 27,012 women at a median baseline age of 48.4 (range: 35.2–65.2) years, and a median BMI of 24.7 (see Table 1, and Additional file 1: Figure S1). Overall, 40.8% of the women were postmenopausal at baseline. Among the postmenopausal women, 46.0% reported to use HT. The average follow-up duration was 13.0 (±3.1) years. Median age at diagnosis among the 657 breast cancer cases was 60.2 (range: 38.9–78.6) years.

**Table 1:** Characteristics of the study population

N	27,012
Age at recruitment^a^	48.4 (41.2, 57.0)
Anthropometric parameters^a^	
BMI (kg/m^2^)	24.7 (22.3, 28.0)
Height (cm)	163.2 (159.0, 167.5)
Menopausal Status	
Pre- and perimenopausal (%)	59.2
Postmenopausal (%)	40.8
Hormone therapy (%)^b^	
User at baseline (%)	46.0
Non-user at baseline (%)	54.0
Number of full-term pregnancies^c^	1.7 (0, 8)
Smoking Status	
Never smokers (%)	55.7
Former smokers (%)	25.6
Current smokers (%)	18.7
Education Level	
University Degree (%)	34.4
No University Degree (%)	65.6

^a^Median values (p25, 75) are shown for continuous variables

^b^Postmenopausal women only

^c^Mean value (Minimum, Maximum)

Tumor stages and grades at diagnosis were as follows; In situ: 7.0%, Stage I: 38.7%, Stage II: 41.0%, Stage III: 11.3%, Stage IV: 2.0%; Grade I: 12.4%, Grade II: 56.8%, Grade III: 30.8% (Additional file 2: Table S1). Of the invasive tumors, 70.5% were carcinoma of no special type (NST), 18.3% lobular carcinoma, and 11.1% other; of the in situ tumors, 67.4% were ductal carcinoma, 13.0% were lobular carcinoma, and 19.6% other (Additional file 2: Table S3). The proportions of subtypes indicating more favorable prognosis were 84.8% for ER+, 70.7% for PR+, 87.5% for HER2-, 83.1% for Ki67_low_, 66.0% for Bcl-2+ and 80.1% for p53-. Frequencies of luminal A (ER+ and/or PR+, HER2- and Ki67_low_), luminal B (ER+ and/or PR+, HER2- and Ki67_high_), Her2+, and triple negative (ER-, PR-, and HER2-) tumors were 68.6, 8.4, 9.7, and 13.3%.

**Fig. 1:**
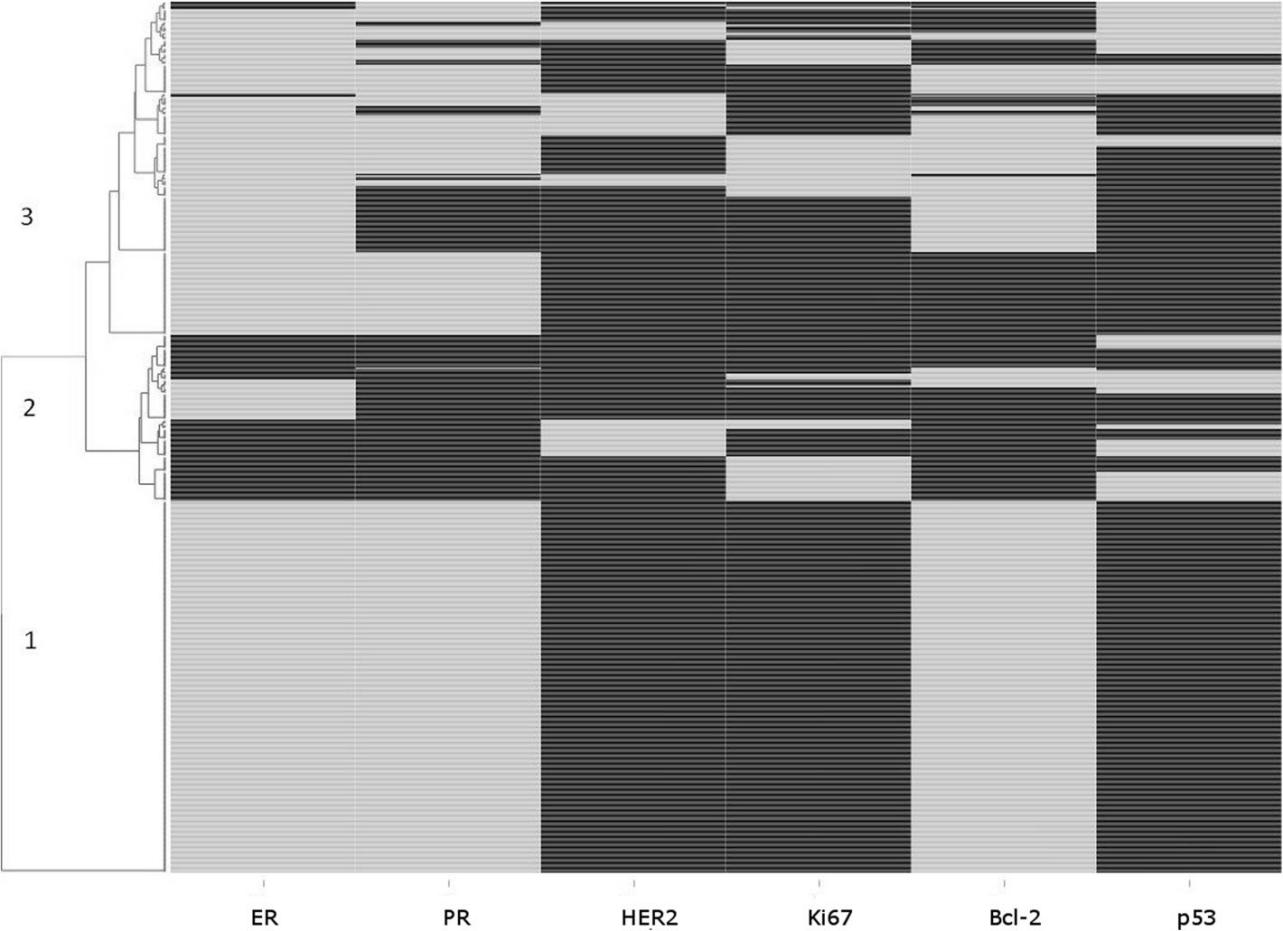
Frequencies of combined tumor subtypes as derived from hierarchical clustering, with the top three clusters marked in the dendrogram; light bars indicate positivity (or high proliferation activity in case of Ki67)

The results of the unsupervised hierarchical clustering of breast cancer cases according to IHC staining profiles are shown in Fig. 1. The three main clusters identified by hierarchical clustering can be characterized as follows: Cluster 1 (42.7% of all cases) contains tumors with a profile of individual markers indicative of low aggressiveness (all cases are ER+, PR+, HER2-, Ki67_low_, Bcl-2+ and p53-). Cluster 2 (19.0% of all cases) contains ER- tumors and ER+ tumors that are Bcl-2 negative. Cluster 3 (38.3% of all cases) mainly contains ER+ tumors that, unlike the ER+ tumors in cluster 1, show at least one criterion pointing to higher aggressiveness (i.e. p53 positivity, Bcl-2 negativity, high Ki67 expression, or HER2 positivity).

### BMI and risk of breast cancer by tumor subtype

Among postmenopausal non-users of HT, BMI was directly associated with higher overall breast cancer risk (HR per 5 kg/m^2^: 1.27 [95% CI: 1.07, 1.50], *p* = 0.005), while a significant inverse association was observed among HT users (HR: 0.80 [0.66, 0.98], *p* = 0.024) (Table 2). BMI was not significantly associated with overall breast cancer risk in pre- and perimenopausal women (HR: 0.98 [0.85, 1.12], *p* = 0.72).

**Table 2:** Hazard ratios of overall breast cancer across tertiles of BMI^a^

	Postmenopausal non-users of HT^b^			Postmenopausal users of HT^b^			Pre- and perimenopausal women^b^		
	Cases (n)	HR	CI (95%)	Cases (n)	HR	CI (95%)	Cases (n)	HR	CI (95%)
Tertile 1	14	1		65	1		141	1	
Tertile 2	43	1.87	(1.00,3.49)	92	0.97	(0.70,1.34)	85	0.76	(0.57,1.00)
Tertile 3	79	2.28	(1.23,4.16)	56	0.69	(0.47,1.00)	82	0.93	(0.70,1.24)
*Per 5 kg/m* ^2^		*1.27*	*(1.07,1.50)*		*0.80*	*(0.66,0.98)*		*0.98*	*(0.85,1.12)*
*p trend*		*0.005*			*0.024*			*0.72*	

Median (p25, p75) values of BMI: Tertile 1: 21.4 (20.4, 22.3), Tertile 2: 24.8 (23.9, 25.7); Tertile 3: 29.9 (28.1, 32.7)

^a^From Cox regression models adjusted for height, number of full-term pregnancies, pill use, education level, smoking status, and study center

^b^At baseline (*HT* hormone therapy)

Analyses stratified by tumor subtypes as derived from hierarchical clustering are shown in Table 3. Among postmenopausal non-users of HT, each 5 kg/m^2^ increment of BMI was directly and significantly associated with the risk of less aggressive cluster 1 tumors, i.e. tumors that were ER+, PR+, HER2-, Ki67_low_, Bcl-2+ and p53-, with a HR per 5 kg/m^2^ of 1.44 [95% CI: 1.10, 1.90], *p* = 0.009). BMI was not associated with more aggressive cluster 2 and cluster 3 tumors (Table 3). Among HT-users, BMI was significantly associated with lower risk of less aggressive cluster 1 tumors (HR per 5 kg/m^2^: 0.68 [0.50, 0.94], *p* = 0.018); again, no significant associations with the risks of more aggressive cluster 2 and cluster 3 tumors were observed. While risk analyses per 5 kg/m^2^ did not reveal significant associations between BMI and risks of any tumor subtype in pre- and perimenopausal women, it is of note that women in the highest BMI tertile showed a significantly lower risk of less aggressive cluster 1 tumors as compared to women in the lowest BMI tertile (HR_Tertile3 vs. Tertile1_: 0.55 [0.33, 0.93]). Sensitivity analyses excluding in situ cases yielded similar highly similar results (Additional file 2: Table S4). Associations between BMI and risk of luminal A tumors were similar to those between BMI and risk of cluster 1 tumors (Additional file 2: Table S5); there were no significant associations with luminal B and triple negative tumors.

**Table 3:** Hazard ratios of breast cancer across tertiles of BMI by clusters of breast tumors from hierarchical clustering (see Fig. 1)^a^

	Postmenopausal non-users of HT^b^				Postmenopausal users of HT^b^				Pre- and perimenopausal women^b^			
		Cases (n)	HR	CI (95%)		Cases (n)	HR	CI (95%)		Cases (n)	HR	CI (95%)
Cluster 1	Tertile 1	4	1		Tertile 1	30	1		Tertile 1	59	1	
*(ER+, PR+, HER2-, Ki67*_*low*_ *, bcl-2+, and p53-)*	Tertile 2	8	1.02	(0.31,3.40)	Tertile 2	32	0.74	(0.44,1.22)	Tertile 2	31	0.64	(0.41,1.00)
	Tertile 3	33	2.50	(0.86,7.23)	Tertile 3	24	0.61	(0.35,1.06)	Tertile 3	21	0.55	(0.33,0.93)
	*Per 5 kg/m* ^2^		*1.44*	*(1.10,1.90)*	*Per 5 kg/m* ^2^		*0.68*	*(0.50,0.94)*	*Per 5 kg/m* ^2^		*0.85*	*(0.67,1.08)*
	*p trend*		*0.009*		*p trend*		*0.018*		*p trend*		*0.19*	
Cluster 2	Tertile 1	5	1		Tertile 1	10	1		Tertile 1	18	1	
*(ER- or ER+ that are Bcl-2-)*	Tertile 2	6	0.77	(0.23,2.56)	Tertile 2	18	1.14	(0.52,2.53)	Tertile 2	9	0.59	(0.26,1.32)
	Tertile 3	16	1.40	(0.49,4.04)	Tertile 3	6	0.43	(0.15,1.21)	Tertile 3	20	1.52	(0.77,3.00)
	*Per 5 kg/m* ^2^		*1.15*	*(0.78,1.70)*	*Per 5 kg/m* ^2^		*0.83*	*(0.52,1.32)*	*Per 5 kg/m* ^2^		*1.22*	*(0.91,1.62)*
	*p trend*		*0.47*		*p trend*		*0.42*		*p trend*		*0.18*	
Cluster 3	Tertile 1	5	1		Tertile 1	20	1		Tertile 1	48	1	
*(ER+ with at least one other marker indicative of higher aggressiveness)*	Tertile 2	21	2.98	(1.01,8.75)	Tertile 2	33	1.20	(0.68,2.12)	Tertile 2	26	0.72	(0.44,1.18)
	Tertile 3	16	1.57	(0.51,4.83)	Tertile 3	17	0.77	(0.39,1.51)	Tertile 3	31	1.13	(0.70,1.82)
	*Per 5 kg/m* ^2^		*1.00*	*(0.71,1.42)*	*Per 5 kg/m* ^2^		*0.82*	*(0.58,1.15)*	*Per 5 kg/m* ^2^		*0.94*	*(0.74,1.19)*
	*p trend*		*0.99*		*p trend*		*0.24*		*p trend*		*0.60*	

Median (p25, p75) values of BMI: Tertile 1: 21.4 (20.4, 22.3), Tertile 2: 24.8 (23.9, 25.7); Tertile 3: 29.9 (28.1, 32.7)

No statistical heterogeneity of HRs across subtypes was observed

^a^From Cox regression models adjusted for height, number of full-term pregnancies, pill use, education level, smoking status, and study center ^b^At baseline (*HT* hormone therapy)

In analyses on breast tumor subtypes defined by individual markers, BMI was significantly positively associated with risk of ER+, PR+, HER2-, Ki67_low_, Bcl-2+ and p53- tumors among postmenopausal non-users of HT (Additional file 2: Table S6). By contrast, no significant associations with ER-, PR-, HER2+, Ki67_high_, Bcl-2- and p53+ tumors were observed. With respect to postmenopausal users of HT, Cox regression analyses showed significant inverse associations with risks of ER+, HER2-, Ki67_low_, Bcl-2+ and p53- tumors, and a non-significant tendency for an inverse association with PR+ breast cancer (Additional file 2: Table S7). Again, there were no significant associations with risk of ER-, PR-, HER2+, Ki67_high_, Bcl-2- and p53+ tumors. Among pre- and perimenopausal women, BMI was not significantly associated with risks of any tumor subtype defined by individual markers (Additional file 2: Table S8). The results on BMI and risks of tumor subtypes defined by individual markers were similar after exclusion of in situ cases (see Additional file 2: Table S9, Table S10, and Table S11).

The directions of associations with risk of tumor subtypes were highly similar when using waist and hip circumference as anthropometric indices of obesity instead of BMI, while the associations between waist-to-hip ratio and breast cancer risk were weaker and non-significant (data not shown). Risk associations among premenopausal women *only* were very similar as the presented associations among *peri- and* premenopausal women (data not shown). Importantly, no formal heterogeneity of associations between anthropometric factors and breast cancer risk across tumor subtypes, as either derived from hierarchical clustering or defined by individual IHC markers, was observed.

## Discussion

Here, we examined associations between BMI and breast cancer risk by tumor subtypes characterized by six immunohistochemical markers. Among postmenopausal women who did not use HT at the time of recruitment, higher BMI was significantly associated with increased risk of less aggressive tumors, as either defined by individual markers (ER+, PR+, HER2-, Ki67_low_, Bcl-2+, p53-) or a combination of these markers derived from hierarchical cluster analysis (cluster 1). By contrast, we observed no significant associations between BMI and risk of more aggressive tumors, irrespective of whether subtype classification was based on single markers or on marker combinations (clusters 2 and 3). Among HT users, higher BMI was linearly associated with reduced relative risk of less aggressive (hormone receptor positive, HER-, Ki67_low_, Bcl-2+, or cluster 1) tumors, while there were no significant associations with more aggressive tumors. Analyses by single markers did not reveal any significant associations among pre- and perimenopausal women, whereas risk of cluster 1 tumors was lower among women in the highest BMI tertile compared to those in the lowest.

Various studies have shown associations between obesity and an increased risk of breast cancer among postmenopausal non-users of HT, particularly of ER+ / PR+ breast cancer, but not ER- / PR- breast cancer [4, 22, 23]. Our present data confirm the association with hormone-receptor positive breast cancer and additionally indicate that postmenopausal obesity may be related to an overall less aggressive molecular subtype of breast cancer characterized by a lower proliferation rate (Ki67_low_), Bcl-2 positivity and p53 negativity – immunohistochemical characteristics that are each associated with better prognosis [12, 24–26]. The inverse overall association between obesity and breast cancer risk among HT users that we observed is in agreement with previous data from the full EPIC-Europe cohort [27]. Our results suggest that this inverse association might be strongest for (if not restricted to) the less aggressive tumor subtypes, which is in contrast, however, with earlier observations in the EPIC-Europe Study, which were suggestive of an inverse association between BMI and breast cancer risk among users of HT for ER- / PR- but not ER+ / PR+ tumors [4]. Thus, and given the lack of further studies on obesity and breast cancer risk by tumor subtypes among HT users [28], the associations observed in the present study require replication. Our observation of a lower risk of less aggressive tumors among pre- and perimenopausal women in the highest BMI tertile is consistent with results of a meta-analysis, in which BMI was significantly inversely associated with the risk of ER+/PR+ tumors but not ER-/PR- tumors in premenopausal women [22].

Biological mechanisms that may underlie the association between obesity and breast cancer include altered sex hormone metabolism, adipokine signaling, subclinical inflammation, hyperglycaemia, hyperinsulinaemia, and increased IGF-1 signaling [15, 29]. Differential associations of obesity and breast cancer risk by hormone receptor status likely reflect a greater responsiveness of ER+ / PR+ tumors to these mechanisms [4, 30]. However, it is largely unknown why obesity should predispose to p53- and Bcl-2+ tumor subtypes in postmenopausal women, as indicated by our data. The expression of p53 in breast adipose stromal cells is downregulated by obesity-induced prostaglandin E_2_ (PGE_2_), which results in a local upregulation of aromatase activity and estrogen production [31], and estrogen receptor has also been demonstrated to downregulate p53 and cause tumor cell proliferation [31, 32]. Bcl-2 proteins, by contrast, have been proposed to exert pro-apoptotic effects [12, 25, 33] and influence p53-mediated cell-death [31, 34]. Thus, ER positivity, Bcl-2 positivity and p53 negativity, which co-occurred in a majority of breast cancer cases in the present analyses, all appear to be part of a more general molecular constellation that could be driven by obesity, even though more experimental insight is needed to better understand the interplay between obesity and these tumor characteristics. In addition, larger epidemiological datasets are needed to stratify ER positive and ER negative tumors by p53 or Bcl-2 status, which was not possible due to sample size restrictions in the present study.

Our findings among postmenopausal non-users of HT might suggest better prognosis in obese breast cancer patients, as they may be more likely to have less aggressive tumor subtypes than lean patients. Yet, prospective analyses in cohorts of breast cancer patients have clearly shown that breast cancer-specific survival is negatively impacted by obesity irrespective of menopausal status or hormone receptor status of the tumor [35, 36]. These paradoxical observations may be explained by lower efficiency of anticancer drugs, particularly aromatase inhibitors, in obese patients and by better compliance to treatment among normal weight patients [37]; still, further studies are needed to resolve the paradox as to why obesity may be related to an increased risk of less aggressive breast tumors, while at the same time being associated with worse prognosis irrespective of the tumor subtype.

Several limitations apply to our study. First, by using TMAs from preserved tumor material to assess tumor subtypes, we ensured homogeneity of testing conditions. However, when compared to full-slice IHC staining done for diagnostic purposes, IHC performed on TMAs may be more prone to misclassification of subtypes, especially when the tumor tissue exhibits heterogeneous expression of the markers in question and visual estimation of positive tumor cells is used. To minimize such misclassification, we used two tissue cores per tumor. Nevertheless, we cannot rule out that misclassification of tumor subtypes diluted associations in our study to some degree. Second, case numbers in our study may have been too low to detect weaker associations in some subgroups, especially for the more rare and aggressive cancer subtypes. Due to lower numbers of these tumors, tests for statistical heterogeneity in the associations between obesity and breast cancer risk across tumor subtypes were limited. In this context, it is worth mentioning that in previous analyses of the full European EPIC cohort, heterogeneity in BMI breast cancer risk associations by ER/PR status was restricted to women older than 65 years at diagnosis [4], and that our sample size was not sufficient to further stratify analyses by age groups. Thus, our main observation – associations of obesity with less aggressive breast cancer subtypes – requires replication in larger-scale studies and pooled analyses. This is also true with regard to further stratification of analyses by histological types of breast cancer and cancer stage (e.g. invasive vs. in situ or ductal vs. lobular), for which case numbers in the present study were not sufficient. Another limitation is that we did not have data on family history of breast cancer for statistical adjustment. Finally, as many similar cohort studies on BMI and breast cancer risk, we could not address changes in weight over time, even though weight changes in our population are moderate according to self-reports [38].

## Conclusion

In the present study, we evaluated associations between obesity and breast cancer risk by tumor subtypes, as defined by six immunohistochemical markers used in clinical routine to guide treatment and determine prognosis. Our data suggests that obesity is related to ER+, PR+, HER2-, Ki67_low_, Bcl-2+ and p53- tumors, i.e. such with lower aggressiveness, in postmenopausal women. Further mechanistic studies are needed to determine which biological mechanisms underlie the detected associations, and larger pooled analyses of prospective cohort data will be required to further investigate relationships between obesity and molecular breast tumor subtypes, and particularly the less frequent subtypes, in more detail.

## Additional files

Additional file 1: **Figure S1.** Flow Chart. (DOCX 29 kb)

Additional file 2: **Table S1.** Characteristics of breast cancer cases with and without available immunohistochemistry (IHC) markers; **Table S2.** Antibodies; **Table S3.** Frequency of histological tumor types; **Table S4.** Hazard ratios of breast cancer across tertiles of BMI by clusters of breast tumors from hierarchical clustering, after exclusion of situ tumors; **Table S5.** Hazard ratios of luminal A breast cancer across tertiles of BMI; **Table S6.** Hazard ratios of breast cancer subtypes across tertiles of BMI among postmenopausal non-users of hormone therapy; **Table S7.** Hazard ratios of breast cancer subtypes across tertiles of BMI among postmenopausal users of hormone therapy; **Table S8.** Hazard ratios of breast cancer subtypes across tertiles of BMI among pre- and perimenopausal women; **Table S9.** Hazard ratios of breast cancer subtypes across tertiles of BMI among postmenopausal non-users of hormone therapy, after exclusion of situ tumors; **Table S10.** Hazard ratios of breast cancer subtypes across tertiles of BMI among postmenopausal users of hormone therapy, after exclusion of situ tumors; **Table S11.** Hazard ratios of breast cancer subtypes across tertiles of BMI among pre- and perimenopausal women, after exclusion of situ tumors. (DOCX 84 kb)

## Abbreviations

Bcl-2: B-cell lymphoma 2
BMI: Body mass index
CI: Confidence interval
EPIC: European Prospective Investigation into Cancer and Nutrition
ER: Estrogen receptor
FFPE: formalin-fixed paraffin-embedded
HER2: Human epidermal growth factor receptor 2
HR: Hazard ratio
HT: Hormone therapy
IGF-1: Insulin-like growth factor 1
IHC: Immunohistochemistry
PR: Progesterone receptor
TMA: Tissue microarray
