# Supplementary material for: Obesity as risk factor for subtypes of breast cancer: results from a prospective cohort study
Source: BMC Cancer. 2018 May 31;18:616. doi: 10.1186/s12885-018-4548-6 (PMC5984403; doi:10.1186/s12885-018-4548-6)
Supplement: Supplementary file 1 — Figure S1. Flow Chart. (DOCX 29 kb) [file 12885_2018_4548_MOESM1_ESM.docx]

Additional file 1: Figure S1 Flow Chart

Prevalent Cancer or Unknown Cancer Status

(n=1,669)

Lost-to-follow-up

(n=947)

Validation of Cancer Status not possible

(n=23)

Missing Covariates

(n=181)

Incident Cases without Tumor Blocks

(n=438)

Pre-and peri-menopausal women (n_total_=15,976; n_cases_=308)

Postmenopausal users of HT

(n_total_=5,072;

n_cases_=213)

Postmenopausal non-users of HT

(n_total_=5,964;

n_cases_=136)

Analytical Sample

(n_total_=27,012; n_cases_=657)

Female Participants of EPIC-Germany

(n=30,270)
