# Supplementary material for: Obesity as risk factor for subtypes of breast cancer: results from a prospective cohort study
Source: BMC Cancer. 2018 May 31;18:616. doi: 10.1186/s12885-018-4548-6 (PMC5984403; doi:10.1186/s12885-018-4548-6)
Supplement: Supplementary file 2 — Table S1. Characteristics of breast cancer cases with and without available immunohistochemistry (IHC) markers; Table S2. Antibodies; Table S3. Frequency of histological tumor types; Table S4. Hazard ratios of breast cancer across tertiles of BMI by clusters of breast tumors from hierarchical clustering, after exclusion of situ tumors; Table S5. Hazard ratios of luminal A breast cancer across tertiles of BMI; Table S6. Hazard ratios of breast cancer subtypes across tertiles of BMI among postmenopausal non-users of hormone therapy; Table S7. Hazard ratios of breast cancer subtypes across tertiles of BMI among postmenopausal users of hormone therapy; Table S8. Hazard ratios of breast cancer subtypes across tertiles of BMI among pre- and perimenopausal women; Table S9. Hazard ratios of breast cancer subtypes across tertiles of BMI among postmenopausal non-users of hormone therapy, after exclusion of situ tumors; Table S10. Hazard ratios of breast cancer subtypes across tertiles of BMI among postmenopausal users of hormone therapy, after exclusion of situ tumors; Table S11. Hazard ratios of breast cancer subtypes across tertiles of BMI among pre- and perimenopausal women, after exclusion of situ tumors. (DOCX 84 kb) [file 12885_2018_4548_MOESM2_ESM.docx]

# Additional file 2

| **Table S1** Characteristics of breast cancer cases with and without available immunohistochemistry (IHC) markers | | | | |
| --- | --- | --- | --- | --- |
|  | *IHC markers available* | *IHC markers not available* | *p* |  |
| N | 657 | 438 |  |  |
| Age at recruitment^1^ | 51.7 ± 8.1 | 51.2 ± 8.3 | 0.65 |  |
| Age at diagnosis^1^ | 59.2 ± 8.6 | 58.4 ± 9.0 | 0.13 |  |
| Anthropometric parameters^1^ |  |  |  |  |
| BMI (kg/m^2^) | 25.7 ± 4.5 | 25.8 ± 4.7 | 0.65 |  |
| Height (cm) | 164.0 ± 6.0 | 163.5 ± 6.5 | 0.23 |  |
| Waist circumference (cm) | 81.6 ± 11.0 | 81.6 ± 11.6 | 0.96 |  |
| Alcohol consumption (g/day) | 9.9 ± 12.4 | 10.4 ± 12.3 | 0.47 |  |
| Postmenopausal women (%) | 53.1 | 50.5 |  |  |
| Hormone therapy users (%)^2^ | 38.2 | 34.5 | 0.21 |  |
| Number of full-term pregnancies | 1.7 ± 1.0 | 1.7 ± 1.1 | 0.89 |  |
| Smoking Status |  |  | 0.82 |  |
| Never smokers (%) | 58.6 | 59.6 |  |  |
| Former smokers (%) | 23.9 | 24.4 |  |  |
| Current smokers (%) | 17.4 | 16.2 |  |  |
| Missing (%) | 0.1 | 0 |  |  |
| Education Level |  |  | 0.62 |  |
| University Degree (%) | 34.9 | 36.4 |  |  |
| No University Degree (%) | 65.1 | 63.4 |  |  |
| Tumor size |  |  | 0.16 |  |
| <2cm | 56.0 | 62.0 |  |  |
| ≥2 cm | 44.0 | 38.0 |  |  |
| Tumor Stage |  |  | <0.01 |  |
| Stage 0 | 7.0 | 15.1 |  |  |
| Stage I | 38.7 | 28.8 |  |  |
| Stage II | 41.0 | 37.7 |  |  |
| Stage III | 11.3 | 16.4 |  |  |
| Stage IV | 2.0 | 2.0 |  |  |
| Tumor Grade |  |  | <0.01 |  |
| Grade I | 12.5 | 20.3 |  |  |
| Grade II | 56.8 | 52.1 |  |  |
| Grade III | 30.7 | 27.6 |  |  |
| ^1^Mean values ± standard deviations are shown for continuous variables  ^2^Postmenopausal women only  P values from T-tests (cont. variables) and Chi2 tests (cat. variables) | | | |  |

| **Table S2** Antibodies | | | | |
| --- | --- | --- | --- | --- |
| *Antibody* | *Clone* | *Pretreatment* | *Dilution* | *Supplier* |
| ER | SP1 | pH 9 | 1:50 | NeoMarkers, Fremont, CA, USA |
| PR | PgR636 | pH 6.1 | 1:100 | Dako, Hamburg, Germany |
| HER2 | poly | pH 6.1 | 1:500 | Dako, Hamburg, Germany |
| Ki67 | 30-9 | pH 9 | 1:200 | Dako, Hamburg, Germany |
| Bcl-2 | 124 | pH 6.1 | 1:75 | Dako, Hamburg, Germany |
| p53 | DO-7 | pH 9 | 1:100 | Dako, Hamburg, Germany |

| **Table S3** Frequency of histological tumor types | | |
| --- | --- | --- |
|  | n | % |
| ***Invasive breast cancer*** |  |  |
| Invasive carcinoma of no special type | 431 | 70.5% |
| Invasive lobular carcinoma | 112 | 18.3% |
| Tubular carcinoma | 18 | 2.9% |
| Carcinoma of mixed type | 17 | 2.8% |
| Cribriform carcinoma | 4 | 0.7% |
| Mucinous carcinoma | 4 | 0.7% |
| Papillary carcinoma | 4 | 0.7% |
| Medullary carcinoma | 4 | 0.7% |
| Invasive Paget's disease | 3 | 0.5% |
| Neuroendocrine carcinoma | 2 | 0.3% |
| Invasive comedocarcinoma | 1 | 0.2% |
| Invasive Phyllodes tumor | 1 | 0.2% |
| Squamous cell carcinoma | 1 | 0.2% |
| Unspecified | 9 | 1.5% |
| ***Noninvasive breast cancer*** |  |  |
| Ductal carcinoma in situ (DCIS) | 31 | 67.4% |
| Lobular carcinoma in situ (LCIS) | 6 | 13.0% |
| Noninvasive comedocarcinoma | 3 | 6.5% |
| Cribriform carcinoma in situ | 2 | 4.3% |
| Papillary ductal carcinoma in situ | 2 | 4.3% |
| Paget's disease in situ | 1 | 2.2% |
| Unspecified | 1 | 2.2% |

| **Table S4** Hazard ratios of breast cancer across tertiles of BMI by clusters of breast tumors from hierarchical clustering (see Figure 1), after exclusion of in situ cases^1^ | | | | | | | | | | | | | | | |
| --- | --- | --- | --- | --- | --- | --- | --- | --- | --- | --- | --- | --- | --- | --- | --- |
|  | **Postmenopausal non-users of HT**^2^ | | | | | **Postmenopausal users of HT**^2^ | | | | | **Pre- and perimenopausal women**^2^ | | | | |
|  |  | Cases (n) | HR | CI (95%) |  |  | Cases (n) | HR | CI (95%) |  |  | Cases (n) | HR | CI (95%) |  |
|  |  |  |  |  |  |  |  |  |  |  |  |  |  |  |  |
| **Cluster 1** | Tertile 1 | 4 | 1 |  |  | Tertile 1 | 17 | 1 |  |  | Tertile 1 | 35 | 1 |  |  |
| *(ER+, PR+, HER2-, Ki67_low_, bcl-2+, and p53-)* | Tertile 2 | 7 | 0.9 | (0.26,3.1) |  | Tertile 2 | 16 | 0.6 | (0.3,1.21) |  | Tertile 2 | 18 | 0.61 | (0.34,1.1) |  |
|  | Tertile 3 | 28 | 2.15 | (0.73,6.3) |  | Tertile 3 | 16 | 0.66 | (0.32,1.34) |  | Tertile 3 | 10 | 0.46 | (0.22,0.96) |  |
|  | *Per 5 kg/m2* |  | 1.48 | (1.1,1.99) |  | *Per 5 kg/m2* |  | 0.7 | (0.47,1.06) |  | *Per 5 kg/m2* |  | 0.85 | (0.62,1.17) |  |
|  | *p trend* |  | 0.009 |  |  | *p trend* |  | 0.09 |  |  | *p trend* |  | 0.31 |  |  |
|  |  |  |  |  |  |  |  |  |  |  |  |  |  |  |  |
| **Cluster 2** | Tertile 1 | 1 | 5 | 1 |  | Tertile 1 | 6 | 1 |  |  | Tertile 1 | 16 | 1 |  |  |
| *(ER- or ER+ that are Bcl-2-)* | Tertile 2 | 2 | 6 | 0.75 |  | Tertile 2 | 13 | 1.2 | (0.45,3.24) |  | Tertile 2 | 4 | 0.29 | (0.1,0.89) |  |
|  | Tertile 3 | 3 | 11 | 0.9 |  | Tertile 3 | 6 | 0.6 | (0.19,1.91) |  | Tertile 3 | 16 | 1.33 | (0.63,2.79) |  |
|  | *Per 5 kg/m2* |  | 1.05 | (0.67,1.65) |  | *Per 5 kg/m2* |  | 0.92 | (0.55,1.54) |  | *Per 5 kg/m2* |  | 1.28 | (0.93,1.75) |  |
|  | *p trend* |  | 0.82 |  |  | *p trend* |  | 0.75 |  |  | *p trend* |  | 0.13 |  |  |
|  |  |  |  |  |  |  |  |  |  |  |  |  |  |  |  |
| **Cluster 3** | Tertile 1 | 4 | 1 |  |  | Tertile 1 | 12 | 1 |  |  | Tertile 1 | 36 | 1 |  |  |
| *(ER+ with at least one other marker indicative of higher aggressiveness)* | Tertile 2 | 15 | 2.88 | (0.83,10.04) |  | Tertile 2 | 24 | 1.42 | (0.69,2.89) |  | Tertile 2 | 17 | 0.65 | (0.36,1.17) |  |
|  | Tertile 3 | 12 | 1.64 | (0.45,5.97) |  | Tertile 3 | 12 | 0.9 | (0.39,2.07) |  | Tertile 3 | 23 | 1.15 | (0.66,2) |  |
|  | *Per 5 kg/m2* |  | 1.09 | (0.75,1.59) |  | *Per 5 kg/m2* |  | 0.81 | (0.53,1.22) |  | *Per 5 kg/m2* |  | 1 | (0.76,1.3) |  |
|  | *p trend* |  | 0.65 |  |  | *p trend* |  | 0.31 |  |  | *p trend* |  | 0.97 |  |  |
|  |  |  |  |  |  |  |  |  |  |  |  |  |  |  |  |
| ^1^From Cox regression models adjusted for height, number of full-term pregnancies, pill use, education level, smoking status, and study center ^2^At baseline (HT: hormone therapy)  *Median (p25, p75) values of BMI: Tertile 1: 21.4 (20.4, 22.3), Tertile 2: 24.8 (23.9, 25.7); Tertile 3: 29.9 (28.1, 32.7);*  No statistical heterogeneity of HRs across subtypes was observed; | | | | | | | | | | | | | | | |

| **Table S5** Hazard ratios of luminal A breast cancer across tertiles of BMI^1^ | | | | | | | | | | | | | | | | |
| --- | --- | --- | --- | --- | --- | --- | --- | --- | --- | --- | --- | --- | --- | --- | --- | --- |
|  | **Postmenopausal non-users of HT**^2^ | | | | | **Postmenopausal users of HT**^2^ | | | | | **Pre- and perimenopausal women**^2^ | | | | |  |
|  |  | Cases (n) | HR | CI (95%) |  |  | Cases (n) | HR | CI (95%) |  |  | Cases (n) | HR | CI (95%) |  |  |
| **Luminal A** | Tertile 1 | 8 | 1 |  |  | Tertile 1 | 49 | 1 |  |  | Tertile 1 | 91 | 1 |  |  |  |
|  | Tertile 2 | 22 | 1.68 | (0.71,3.95) |  | Tertile 2 | 63 | 0.91 | (0.62,1.33) |  | Tertile 2 | 55 | 0.76 | (0.54,1.07) |  |  |
|  | Tertile 3 | 51 | 2.49 | (1.11,5.58) |  | Tertile 3 | 42 | 0.71 | (0.47,1.09) |  | Tertile 3 | 37 | 0.67 | (0.45,0.99) |  |  |
|  | *Per 5 kg/m2* |  | *1.27* | *(1.02,1.58)* |  | *Per 5 kg/m2* |  | *0.78* | *(0.62,0.98)* |  | *Per 5 kg/m2* |  | *0.86* | *(0.71,1.04)* |  |  |
|  | *p trend* |  | *0.035* |  |  | *p trend* |  | *0.031* |  |  | *p trend* |  | *0.11* |  |  |  |
|  |  |  |  |  |  |  |  |  |  |  |  |  |  |  |  |  |
| ^1^From Cox regression models adjusted for height, number of full-term pregnancies, pill use, education level, smoking status, and study center ^2^At baseline (HT: hormone therapy)  *Median (p25, p75) values of BMI: Tertile 1: 21.4 (20.4, 22.3), Tertile 2: 24.8 (23.9, 25.7); Tertile 3: 29.9 (28.1, 32.7);* | | | | | | | | | | | | | | | |  |

| **Table S6** Hazard ratios of breast cancer subtypes across tertiles of BMI among postmenopausal non-users of hormone therapy^1^ | | | | | | | | | | |
| --- | --- | --- | --- | --- | --- | --- | --- | --- | --- | --- |
|  | Tertile | n cases | HR | (95 % CI) |  |  | Tertile | n cases | HR | (95 % CI) |
| **ER+** | 1 | 9 | 1 |  |  | **ER-** | 1 | 5 | 1 |  |
|  | 2 | 36 | 2.49 | (1.15,5.39) |  |  | 2 | 6 | 0.75 | (0.22,2.48) |
|  | 3 | 66 | 3.08 | (1.45,6.51) |  |  | 3 | 11 | 0.84 | (0.27,2.60) |
|  | *Per 5 kg/m^2^* |  | *1.31* | *(1.09,1.58)* |  |  | *Per 5 kg/m^2^* |  | *1.06* | *(0.68,1.64)* |
|  | *p for trend* |  | *0.004* |  |  |  | *p for trend* |  | *0.81* |  |
|  |  |  |  |  |  |  |  |  |  |  |
| **PR+** | 1 | 8 | 1 |  |  | **PR-** | 1 | 6 | 1 |  |
|  | 2 | 22 | 1.49 | (0.66,3.36) |  |  | 2 | 19 | 2.36 | (0.88,6.38) |
|  | 3 | 51 | 2.21 | (1.03,4.76) |  |  | 3 | 25 | 2.16 | (0.80,5.81) |
|  | *Per 5 kg/m^2^* |  | *1.33* | *(1.07,1.64)* |  |  | *Per 5 kg/m^2^* |  | *1.16* | *(0.87,1.56)* |
|  | *p for trend* |  | *0.01* |  |  |  | *p for trend* |  | *0.31* |  |
|  |  |  |  |  |  |  |  |  |  |  |
| **HER2-** | 1 | 12 | 1 |  |  | **HER2+** | 1 | 2 | 1 |  |
|  | 2 | 32 | 1.60 | (0.80,3.19) |  |  | 2 | 9 | 3.29 | (0.69,15.76) |
|  | 3 | 71 | 2.29 | (1.19,4.39) |  |  | 3 | 5 | 1.45 | (0.26,8.03) |
|  | *Per 5 kg/m^2^* |  | *1.33* | *(1.11,1.59)* |  |  | *Per 5 kg/m^2^* |  | *0.82* | *(0.46,1.47)* |
|  | *p for trend* |  | *0.002* |  |  |  | *p for trend* |  | *0.52* |  |
|  |  |  |  |  |  |  |  |  |  |  |
| **Ki67_low_** | 1 | 10 | 1 |  |  | **Ki67_high_** | 1 | 4 | 1 |  |
|  | 2 | 31 | 1.92 | (0.91,4.05) |  |  | 2 | 9 | 1.39 | (0.42,4.57) |
|  | 3 | 57 | 2.32 | (1.12,4.77) |  |  | 3 | 15 | 1.56 | (0.49,4.94) |
|  | *Per 5 kg/m^2^* |  | *1.23* | *(1.01,1.51)* |  |  | *Per 5 kg/m^2^* |  | *1.13* | *(0.77,1.68)* |
|  | *p for trend* |  | *0.043* |  |  |  | *p for trend* |  | *0.53* |  |
|  |  |  |  |  |  |  |  |  |  |  |
| **BCL2+** | 1 | 7 | 1 |  |  | **BCL2-** | 1 | 7 | 1 |  |
|  | 2 | 28 | 2.54 | (1.05,6.17) |  |  | 2 | 11 | 0.93 | (0.36,2.41) |
|  | 3 | 50 | 2.93 | (1.23,6.97) |  |  | 3 | 25 | 1.40 | (0.58,3.35) |
|  | *Per 5 kg/m^2^* |  | *1.26* | *(1.01,1.56)* |  |  | *Per 5 kg/m^2^* |  | *1.16* | *(0.86,1.57)* |
|  | *p for trend* |  | *0.039* |  |  |  | *p for trend* |  | *0.34* |  |
|  |  |  |  |  |  |  |  |  |  |  |
| **P53-** | 1 | 9 | 1 |  |  | **P53+** | 1 | 5 | 1 |  |
|  | 2 | 31 | 2.15 | (0.98,4.69) |  |  | 2 | 9 | 1.01 | (0.34,3.07) |
|  | 3 | 57 | 2.59 | (1.21,5.54) |  |  | 3 | 15 | 1.05 | (0.36,3.01) |
|  | *Per 5 kg/m^2^* |  | *1.24* | *(1.01,1.52)* |  |  | *Per 5 kg/m^2^* |  | *1.09* | *(0.76,1.58)* |
|  | *p for trend* |  | *0.041* |  |  |  | *p for trend* |  | *0.63* |  |
|  |  |  |  |  |  |  |  |  |  |  |
| ^1^From Cox regression models adjusted for height, number of full-term pregnancies, pill use, education level, smoking status, and study center  *Median (p25, p75) values of BMI: Tertile 1: 21.4 (20.4, 22.3), Tertile 2: 24.8 (23.9, 25.7); Tertile 3: 29.9 (28.1, 32.7)* | | | | | | | | | | |

| **Table S7** Hazard ratios of breast cancer subtypes across tertiles of BMI among postmenopausal users of hormone therapy^1^ | | | | | | | | | | |
| --- | --- | --- | --- | --- | --- | --- | --- | --- | --- | --- |
|  | Tertile | n cases | HR | (95 % CI) |  |  | Tertile | n cases | HR | (95 % CI) |
| **ER+** | 1 | 60 | 1 |  |  | **ER-** | 1 | 5 | 1 |  |
|  | 2 | 76 | 0.89 | (0.63,1.25) |  |  | 2 | 16 | 1.91 | (0.69,5.31) |
|  | 3 | 50 | 0.68 | (0.46,1.01) |  |  | 3 | 6 | 0.78 | (0.23,2.63) |
|  | *Per 5 kg/m^2^* |  | *0.77* | *(0.63,0.95)* |  |  | *Per 5 kg/m^2^* |  | *0.99* | *(0.61,1.61)* |
|  | *p for trend* |  | *0.016* |  |  |  | *p for trend* |  | *0.96* |  |
|  |  |  |  |  |  |  |  |  |  |  |
| **PR+** | 1 | 48 | 1 |  |  | **PR-** | 1 | 15 | 1 |  |
|  | 2 | 62 | 0.89 | (0.61,1.31) |  |  | 2 | 29 | 1.35 | (0.71,2.56) |
|  | 3 | 45 | 0.74 | (0.49,1.14) |  |  | 3 | 10 | 0.54 | (0.24,1.24) |
|  | *Per 5 kg/m^2^* |  | *0.80* | *(0.64,1.00)* |  |  | *Per 5 kg/m^2^* |  | *0.84* | *(0.58,1.23)* |
|  | *p for trend* |  | *0.051* |  |  |  | *p for trend* |  | *0.38* |  |
|  |  |  |  |  |  |  |  |  |  |  |
| **HER2-** | 1 | 59 | 1 |  |  | **HER2+** | 1 | 6 | 1 |  |
|  | 2 | 82 | 0.97 | (0.69,1.37) |  |  | 2 | 10 | 0.99 | (0.35,2.76) |
|  | 3 | 48 | 0.68 | (0.45,1.00) |  |  | 3 | 8 | 0.79 | (0.27,2.36) |
|  | *Per 5 kg/m^2^* |  | *0.80* | *(0.65,0.98)* |  |  | *Per 5 kg/m^2^* |  | *0.80* | *(0.47,1.39)* |
|  | *p for trend* |  | *0.035* |  |  |  | *p for trend* |  | *0.43* |  |
|  |  |  |  |  |  |  |  |  |  |  |
| **Ki67_low_** | 1 | 53 | 1 |  |  | **Ki67_high_** | 1 | 10 | 1 |  |
|  | 2 | 78 | 1.02 | (0.71,1.46) |  |  | 2 | 9 | 0.55 | (0.21,1.41) |
|  | 3 | 49 | 0.75 | (0.50,1.13) |  |  | 3 | 4 | 0.29 | (0.09,0.96) |
|  | *Per 5 kg/m^2^* |  | *0.79* | *(0.64,0.98)* |  |  | *Per 5 kg/m^2^* |  | *0.73* | *(0.41,1.33)* |
|  | *p for trend* |  | *0.030* |  |  |  | *p for trend* |  | *0.31* |  |
|  |  |  |  |  |  |  |  |  |  |  |
| **BCL2+** | 1 | 42 | 1 |  |  | **BCL2-** | 1 | 21 | 1 |  |
|  | 2 | 53 | 0.85 | (0.56,1.29) |  |  | 2 | 32 | 1.06 | (0.60,1.86) |
|  | 3 | 36 | 0.66 | (0.41,1.04) |  |  | 3 | 16 | 0.65 | (0.33,1.27) |
|  | *Per 5 kg/m^2^* |  | *0.77* | *(0.60,0.98)* |  |  | *Per 5 kg/m^2^* |  | *0.82* | *(0.59,1.15)* |
|  | *p for trend* |  | *0.037* |  |  |  | *p for trend* |  | *0.25* |  |
|  |  |  |  |  |  |  |  |  |  |  |
| **P53-** | 1 | 47 | 1 |  |  | **P53+** | 1 | 17 | 1 |  |
|  | 2 | 67 | 1 | (0.69,1.47) |  |  | 2 | 22 | 0.82 | (0.43,1.58) |
|  | 3 | 43 | 0.75 | (0.49,1.16) |  |  | 3 | 8 | 0.35 | (0.15,0.84) |
|  | *Per 5 kg/m^2^* |  | *0.78* | *(0.62,0.98)* |  |  | *Per 5 kg/m^2^* |  | *0.71* | *(0.47,1.09)* |
|  | *p for trend* |  | *0.031* |  |  |  | *p for trend* |  | *0.12* |  |
|  |  |  |  |  |  |  |  |  |  |  |
| ^1^From Cox regression models adjusted for height, number of full-term pregnancies, pill use, education level, smoking status, and study center  *Median (p25, p75) values of BMI: Tertile 1: 21.4 (20.4, 22.3), Tertile 2: 24.8 (23.9, 25.7); Tertile 3: 29.9 (28.1, 32.7)* | | | | | | | | | | |

| **Table S8** Hazard ratios of breast cancer subtypes across tertiles of BMI among pre- and perimenopausal women^1^ | | | | | | | | | | |
| --- | --- | --- | --- | --- | --- | --- | --- | --- | --- | --- |
|  | Tertile | n cases | HR | (95 % CI) |  |  | Tertile | n cases | HR | (95 % CI) |
| **ER+** | 1 | 116 | 1 |  |  | **ER-** | 1 | 20 | 1 |  |
|  | 2 | 73 | 0.79 | (0.59,1.08) |  |  | 2 | 8 | 0.46 | (0.20,1.06) |
|  | 3 | 60 | 0.85 | (0.62,1.18) |  |  | 3 | 21 | 1.44 | (0.75,2.76) |
|  | *Per 5 kg/m^2^* |  | *0.95* | *(0.81,1.10)* |  |  | *Per 5 kg/m^2^* |  | *1.17* | *(0.87,1.57)* |
|  | *p for trend* |  | *0.48* |  |  |  | *p for trend* |  | *0.30* |  |
|  |  |  |  |  |  |  |  |  |  |  |
| **PR+** | 1 | 105 | 1 |  |  | **PR-** | 1 | 32 | 1 |  |
|  | 2 | 63 | 0.76 | (0.56,1.05) |  |  | 2 | 18 | 0.66 | (0.37,1.18) |
|  | 3 | 47 | 0.75 | (0.53,1.08) |  |  | 3 | 33 | 1.46 | (0.87,2.45) |
|  | *Per 5 kg/m^2^* |  | *0.93* | *(0.79,1.10)* |  |  | *Per 5 kg/m^2^* |  | *1.09* | *(0.86,1.39)* |
|  | *p for trend* |  | *0.40* |  |  |  | *p for trend* |  | *0.47* |  |
|  |  |  |  |  |  |  |  |  |  |  |
| **HER2-** | 1 | 123 | 1 |  |  | **HER2+** | 1 | 18 | 1 |  |
|  | 2 | 73 | 0.74 | (0.55,0.99) |  |  | 2 | 8 | 0.63 | (0.27,1.48) |
|  | 3 | 65 | 0.84 | (0.61,1.15) |  |  | 3 | 15 | 1.51 | (0.72,3.16) |
|  | *Per 5 kg/m^2^* |  | *0.95* | *(0.82,1.10)* |  |  | *Per 5 kg/m^2^* |  | *1.12* | *(0.80,1.56)* |
|  | *p for trend* |  | *0.47* |  |  |  | *p for trend* |  | *0.52* |  |
|  |  |  |  |  |  |  |  |  |  |  |
| **Ki67_low_** | 1 | 113 | 1 |  |  | **Ki67_high_** | 1 | 23 | 1 |  |
|  | 2 | 66 | 0.74 | (0.54,1.01) |  |  | 2 | 10 | 0.53 | (0.25,1.12) |
|  | 3 | 56 | 0.80 | (0.57,1.12) |  |  | 3 | 20 | 1.33 | (0.71,2.51) |
|  | *Per 5 kg/m^2^* |  | *0.91* | *(0.78,1.07)* |  |  | *Per 5 kg/m^2^* |  | *1.13* | *(0.85,1.50)* |
|  | *p for trend* |  | *0.24* |  |  |  | *p for trend* |  | *0.41* |  |
|  |  |  |  |  |  |  |  |  |  |  |
| **BCL2+** | 1 | 98 | 1 |  |  | **BCL2-** | 1 | 37 | 1 |  |
|  | 2 | 53 | 0.69 | (0.49,0.97) |  |  | 2 | 25 | 0.82 | (0.49,1.38) |
|  | 3 | 42 | 0.72 | (0.49,1.05) |  |  | 3 | 37 | 1.47 | (0.90,2.38) |
|  | *Per 5 kg/m^2^* |  | *0.87* | *(0.72,1.04)* |  |  | *Per 5 kg/m^2^* |  | *1.16* | *(0.94,1.43)* |
|  | *p for trend* |  | *0.12* |  |  |  | *p for trend* |  | *0.16* |  |
|  |  |  |  |  |  |  |  |  |  |  |
| **P53-** | 1 | 110 | 1 |  |  | **P53+** | 1 | 23 | 1 |  |
|  | 2 | 65 | 0.72 | (0.53,0.99) |  |  | 2 | 8 | 0.44 | (0.19,1.01) |
|  | 3 | 62 | 0.89 | (0.64,1.23) |  |  | 3 | 15 | 1.05 | (0.53,2.10) |
|  | *Per 5 kg/m^2^* |  | *0.96* | *(0.82,1.12)* |  |  | *Per 5 kg/m^2^* |  | *1.01* | *(0.72,1.42)* |
|  | *p for trend* |  | *0.59* |  |  |  | *p for trend* |  | *0.94* |  |
|  |  |  |  |  |  |  |  |  |  |  |
| ^1^From Cox regression models adjusted for height, number of full-term pregnancies, pill use, education level, smoking status, and study center  *Median (p25, p75) values of BMI: Tertile 1: 21.4 (20.4, 22.3), Tertile 2: 24.8 (23.9, 25.7); Tertile 3: 29.9 (28.1, 32.7)* | | | | | | | | | | |

| **Table S9** Hazard ratios of breast cancer subtypes across tertiles of BMI among postmenopausal non-users of hormone therapy, after exclusion of situ tumors^1^ | | | | | | | | | | |
| --- | --- | --- | --- | --- | --- | --- | --- | --- | --- | --- |
|  | Tertile | n cases | HR | (95 % CI) |  |  | Tertile | n cases | HR | (95 % CI) |
| **ER+** | 1 | 9 | 1 |  |  | **ER-** | 1 | 5 | 1 |  |
|  | 2 | 35 | 2.38 | (1.1,5.16) |  |  | 2 | 5 | 0.62 | (0.18,2.17) |
|  | 3 | 63 | 2.83 | (1.34,6.01) |  |  | 3 | 10 | 0.74 | (0.23,2.34) |
|  | *Per 5 kg/m2* | | *1.28* | *(1.06,1.55)* |  |  | *Per 5 kg/m2* | | *1.02* | *(0.64,1.63)* |
|  | *p for trend* | | *0.01* |  |  |  | *p for trend* | | *0.93* |  |
|  |  |  |  |  |  |  |  |  |  |  |
| **PR+** | 1 | 8 | 1 |  |  | **PR-** | 1 | 6 | 1 |  |
|  | 2 | 21 | 1.41 | (0.62,3.2) |  |  | 2 | 18 | 2.21 | (0.81,6.02) |
|  | 3 | 50 | 2.12 | (0.98,4.57) |  |  | 3 | 22 | 1.83 | (0.67,5.01) |
|  | *Per 5 kg/m2* | | *1.31* | *(1.06,1.63)* |  |  | *Per 5 kg/m2* | | *1.11* | *(0.82,1.52)* |
|  | *p for trend* | | *0.014* |  |  |  | *p for trend* | | *0.50* |  |
|  |  |  |  |  |  |  |  |  |  |  |
| **HER2-** | 1 | 12 | 1 |  |  | **HER2+** | 1 | 2 | 1 |  |
|  | 2 | 32 | 1.6 | (0.8,3.18) |  |  | 2 | 8 | 2.88 | (0.59,14.05) |
|  | 3 | 69 | 2.19 | (1.14,4.22) |  |  | 3 | 4 | 1.13 | (0.19,6.67) |
|  | *Per 5 kg/m2* | | *1.31* | *(1.09,1.57)* |  |  | *Per 5 kg/m2* | | *0.75* | *(0.39,1.42)* |
|  | *p for trend* | | *0.004* |  |  |  | *p for trend* | | *0.37* |  |
|  |  |  |  |  |  |  |  |  |  |  |
| **Ki67_low_** | 1 | 10 | 1 |  |  | **Ki67_high_** | 1 | 4 | 1 |  |
|  | 2 | 28 | 1.69 | (0.79,3.59) |  |  | 2 | 9 | 1.42 | (0.43,4.65) |
|  | 3 | 54 | 2.09 | (1.01,4.32) |  |  | 3 | 15 | 1.58 | (0.5,5.01) |
|  | *Per 5 kg/m2* | | *1.21* | *(0.99,1.5)* |  |  | *Per 5 kg/m2* | | *1.14* | *(0.77,1.68)* |
|  | *p for trend* | | *0.069* |  |  |  | *p for trend* | | *0.52* |  |
|  |  |  |  |  |  |  |  |  |  |  |
| **BCL2+** | 1 | 7 | 1 |  |  | **BCL2-** | 1 | 7 | 1 |  |
|  | 2 | 26 | 2.3 | (0.94,5.61) |  |  | 2 | 11 | 0.91 | (0.35,2.38) |
|  | 3 | 48 | 2.68 | (1.12,6.37) |  |  | 3 | 24 | 1.3 | (0.54,3.14) |
|  | *Per 5 kg/m2* | | *1.24* | *(0.99,1.55)* |  |  | *Per 5 kg/m2* | | *1.13* | *(0.83,1.54)* |
|  | *p for trend* | | *0.056* |  |  |  | *p for trend* | | *0.44* |  |
|  |  |  |  |  |  |  |  |  |  |  |
| **P53-** | 1 | 9 | 1 |  |  | **P53+** | 1 | 5 | 1 |  |
|  | 2 | 28 | 1.9 | (0.86,4.18) |  |  | 2 | 9 | 1.01 | (0.33,3.06) |
|  | 3 | 55 | 2.41 | (1.12,5.15) |  |  | 3 | 14 | 0.95 | (0.33,2.76) |
|  | *Per 5 kg/m2* | | *1.23* | *(1,1.52)* |  |  | *Per 5 kg/m2* | | *1.05* | *(0.72,1.54)* |
|  | *p for trend* | | *0.051* |  |  |  | *p for trend* | | *0.78* |  |
|  |  |  |  |  |  |  |  |  |  |  |
| ^1^From Cox regression models adjusted for height, number of full-term pregnancies, pill use, education level, smoking status, and study center  *Median (p25, p75) values of BMI: Tertile 1: 21.4 (20.4, 22.3), Tertile 2: 24.8 (23.9, 25.7); Tertile 3: 29.9 (28.1, 32.7)* | | | | | | | | | | |

| **Table S10** Hazard ratios of breast cancer subtypes across tertiles of BMI among postmenopausal users of hormone therapy, after exclusion of situ tumors^1^ | | | | | | | | | | |
| --- | --- | --- | --- | --- | --- | --- | --- | --- | --- | --- |
|  | Tertile | n cases | HR | (95 % CI) |  |  | Tertile | n cases | HR | (95 % CI) |
| **ER+** | 1 | 53 | 1 |  |  | **ER-** | 1 | 4 | 1 |  |
|  | 2 | 71 | 0.94 | (0.65,1.35) |  |  | 2 | 15 | 2.1 | (0.69,6.45) |
|  | 3 | 46 | 0.71 | (0.47,1.08) |  |  | 3 | 5 | 0.77 | (0.2,2.94) |
|  | *Per 5 kg/m2* | | *0.79* | *(0.63,0.98)* |  |  | *Per 5 kg/m2* | | *0.97* | *(0.58,1.63)* |
|  | *p for trend* | | *0.03* |  |  |  | *p for trend* | | *0.91* |  |
|  |  |  |  |  |  |  |  |  |  |  |
| **PR+** | 1 | 43 | 1 |  |  | **PR-** | 1 | 12 | 1 |  |
|  | 2 | 57 | 0.92 | (0.62,1.38) |  |  | 2 | 28 | 1.55 | (0.78,3.1) |
|  | 3 | 41 | 0.77 | (0.49,1.21) |  |  | 3 | 9 | 0.58 | (0.24,1.41) |
|  | *Per 5 kg/m2* | | *0.81* | *(0.64,1.02)* |  |  | *Per 5 kg/m2* | | *0.86* | *(0.58,1.26)* |
|  | *p for trend* | | *0.079* |  |  |  | *p for trend* | | *0.44* |  |
|  |  |  |  |  |  |  |  |  |  |  |
| **HER2-** | 1 | 53 | 1 |  |  | **HER2+** | 1 | 4 | 1 |  |
|  | 2 | 78 | 1.02 | (0.71,1.46) |  |  | 2 | 8 | 1.12 | (0.33,3.75) |
|  | 3 | 45 | 0.7 | (0.46,1.06) |  |  | 3 | 6 | 0.88 | (0.24,3.19) |
|  | *Per 5 kg/m2* | | *0.81* | *(0.66,1)* |  |  | *Per 5 kg/m2* | | *0.83* | *(0.44,1.56)* |
|  | *p for trend* | | *0.05* |  |  |  | *p for trend* | | *0.57* |  |
|  |  |  |  |  |  |  |  |  |  |  |
| **Ki67_low_** | 1 | 47 | 1 |  |  | **Ki67_high_** | 1 | 9 | 1 |  |
|  | 2 | 73 | 1.07 | (0.74,1.56) |  |  | 2 | 9 | 0.59 | (0.22,1.55) |
|  | 3 | 46 | 0.79 | (0.52,1.21) |  |  | 3 | 4 | 0.3 | (0.09,1.02) |
|  | *Per 5 kg/m2* | | *0.81* | *(0.65,1)* |  |  | *Per 5 kg/m2* | | *0.74* | *(0.41,1.36)* |
|  | *p for trend* | | *0.054* |  |  |  | *p for trend* | | *0.34* |  |
|  |  |  |  |  |  |  |  |  |  |  |
| **BCL2+** | 1 | 39 | 1 |  |  | **BCL2-** | 1 | 17 | 1 |  |
|  | 2 | 49 | 0.85 | (0.55,1.3) |  |  | 2 | 31 | 1.24 | (0.68,2.28) |
|  | 3 | 33 | 0.64 | (0.4,1.04) |  |  | 3 | 16 | 0.78 | (0.39,1.59) |
|  | *Per 5 kg/m2* | | *0.77* | *(0.6,1)* |  |  | *Per 5 kg/m2* | | *0.86* | *(0.61,1.21)* |
|  | *p for trend* | | *0.049* |  |  |  | *p for trend* | | *0.39* |  |
|  |  |  |  |  |  |  |  |  |  |  |
| **P53-** | 1 | 45 | 1 |  |  | **P53+** | 1 | 12 | 1 |  |
|  | 2 | 61 | 0.95 | (0.64,1.41) |  |  | 2 | 22 | 1.14 | (0.56,2.35) |
|  | 3 | 40 | 0.73 | (0.47,1.14) |  |  | 3 | 8 | 0.48 | (0.19,1.21) |
|  | *Per 5 kg/m2* | | *0.78* | *(0.62,0.99)* |  |  | *Per 5 kg/m2* | | *0.78* | *(0.51,1.21)* |
|  | *p for trend* | | *0.037* |  |  |  | *p for trend* | | *0.27* |  |
|  |  |  |  |  |  |  |  |  |  |  |
| ^1^From Cox regression models adjusted for height, number of full-term pregnancies, pill use, education level, smoking status, and study center  *Median (p25, p75) values of BMI: Tertile 1: 21.4 (20.4, 22.3), Tertile 2: 24.8 (23.9, 25.7); Tertile 3: 29.9 (28.1, 32.7)* | | | | | | | | | | |

| **Table S11** Hazard ratios of breast cancer subtypes across tertiles of BMI among pre- and perimenopausal women, after exclusion of situ tumors ^1^ | | | | | | | | | | |
| --- | --- | --- | --- | --- | --- | --- | --- | --- | --- | --- |
|  | Tertile | n cases | HR | (95 % CI) |  |  | Tertile | n cases | HR | (95 % CI) |
| ER+ | 1 | 110 | 1 |  |  | ER- | 1 | 20 | 1 |  |
|  | 2 | 66 | 0.75 | (0.55,1.03) |  |  | 2 | 7 | 0.4 | (0.17,0.94) |
|  | 3 | 57 | 0.86 | (0.61,1.2) |  |  | 3 | 20 | 1.32 | (0.68,2.56) |
|  | *Per 5 kg/m2* | | *0.96* | *(0.83,1.13)* |  |  | *Per 5 kg/m2* | | *1.15* | *(0.85,1.56)* |
|  | *p for trend* | | *0.64* |  |  |  | *p for trend* | | *0.38* |  |
|  |  |  |  |  |  |  |  |  |  |  |
| PR+ | 1 | 98 | 1 |  |  | PR- | 1 | 32 | 1 |  |
|  | 2 | 57 | 0.72 | (0.52,1.01) |  |  | 2 | 17 | 0.63 | (0.35,1.14) |
|  | 3 | 45 | 0.76 | (0.52,1.1) |  |  | 3 | 31 | 1.39 | (0.82,2.36) |
|  | *Per 5 kg/m2* | | *0.95* | *(0.8,1.12)* |  |  | *Per 5 kg/m2* | | *1.09* | *(0.85,1.39)* |
|  | *p for trend* | | *0.53* |  |  |  | *p for trend* | | *0.50* |  |
|  |  |  |  |  |  |  |  |  |  |  |
| HER2- | 1 | 116 | 1 |  |  | HER2+ | 1 | 18 | 1 |  |
|  | 2 | 64 | 0.68 | (0.49,0.92) |  |  | 2 | 8 | 0.62 | (0.26,1.45) |
|  | 3 | 63 | 0.86 | (0.63,1.19) |  |  | 3 | 13 | 1.27 | (0.59,2.73) |
|  | *Per 5 kg/m2* | | *0.97* | *(0.83,1.13)* |  |  | *Per 5 kg/m2* | | *1.07* | *(0.75,1.53)* |
|  | *p for trend* | | *0.67* |  |  |  | *p for trend* | | *0.71* |  |
|  |  |  |  |  |  |  |  |  |  |  |
| **Ki67_low_** | 1 | 108 | 1 |  |  | **Ki67_high_** | 1 | 22 | 1 |  |
|  | 2 | 60 | 0.7 | (0.51,0.96) |  |  | 2 | 9 | 0.49 | (0.22,1.08) |
|  | 3 | 52 | 0.78 | (0.55,1.1) |  |  | 3 | 20 | 1.36 | (0.71,2.57) |
|  | *Per 5 kg/m2* | | *0.91* | *(0.77,1.08)* |  |  | *Per 5 kg/m2* | | *1.14* | *(0.86,1.53)* |
|  | *p for trend* | | *0.27* |  |  |  | *p for trend* | | *0.37* |  |
|  |  |  |  |  |  |  |  |  |  |  |
| BCL2+ | 1 | 91 | 1 |  |  | BCL2- | 1 | 37 | 1 |  |
|  | 2 | 46 | 0.63 | (0.44,0.91) |  |  | 2 | 24 | 0.78 | (0.46,1.32) |
|  | 3 | 39 | 0.72 | (0.49,1.06) |  |  | 3 | 36 | 1.4 | (0.86,2.28) |
|  | *Per 5 kg/m2* | | *0.88* | *(0.73,1.07)* |  |  | *Per 5 kg/m2* | | *1.15* | *(0.93,1.42)* |
|  | *p for trend* | | *0.20* |  |  |  | *p for trend* | | *0.20* |  |
|  |  |  |  |  |  |  |  |  |  |  |
| P53- | 1 | 106 | 1 |  |  | P53+ | 1 | 22 | 1 |  |
|  | 2 | 58 | 0.66 | (0.48,0.92) |  |  | 2 | 8 | 0.46 | (0.2,1.05) |
|  | 3 | 59 | 0.88 | (0.63,1.23) |  |  | 3 | 15 | 1.08 | (0.54,2.17) |
|  | *Per 5 kg/m2* | | *0.97* | *(0.83,1.13)* |  |  | *Per 5 kg/m2* | | *1.02* | *(0.73,1.43)* |
|  | *p for trend* | | *0.68* |  |  |  | *p for trend* | | *0.91* |  |
|  |  |  |  |  |  |  |  |  |  |  |
| ^1^From Cox regression models adjusted for height, number of full-term pregnancies, pill use, education level, smoking status, and study center  *Median (p25, p75) values of BMI: Tertile 1: 21.4 (20.4, 22.3), Tertile 2: 24.8 (23.9, 25.7); Tertile 3: 29.9 (28.1, 32.7)* | | | | | | | | | | |
